# Affordances and Landscapes: Overcoming the Nature–Culture Dichotomy through Niche Construction Theory

**DOI:** 10.3389/fpsyg.2017.02294

**Published:** 2018-01-09

**Authors:** Manuel Heras-Escribano, Manuel De Pinedo-García

**Affiliations:** ^1^Facultad de Filosofía y Humanidades, Alberto Hurtado University, Santiago, Chile; ^2^Facultad de Filosofía y Letras, Filosofía I, University of Granada, Granada, Spain

**Keywords:** affordance, nice construction, landscape, nature, culture

## Abstract

In this paper we reject the nature–culture dichotomy by means of the idea of affordance or possibility for action, which has important implications for landscape theory. Our hypothesis is that, just as the idea of affordance can serve to overcome the subjective–objective dichotomy, the ideas of landscape and ecological niche, properly defined, would allow us to also transcend the nature–culture dichotomy. First, we introduce an overview of landscape theory, emphasizing processual landscape theory as the most suitable approach for satisfying both cultural and naturalist approaches. After that, we introduce the idea of affordance and we analyze a tension between sociocultural and transcultural affordances (affordances that depend on cultural conventions and affordances that depend on lawful information and bodily aspects of agents). This tension has various implications for landscape theory and ecological niches. Our proposal is that sociocultural and transcultural aspects of affordances could be systematically accommodated if we apply niche construction theory (the theory that explains the process by which organisms modify their selective environments) as a methodological framework for explaining the emergence of ecological niches. This approach will lead us to an integrative account of landscapes as the products of the interaction between human and environmental elements, making it a clear example of a concept that transcends the nature–culture dichotomy.

## Introduction

In this paper we reject the nature–culture dichotomy by means of the idea of affordance, which has important implications for landscape theory. We propose that this can be achieved by applying niche construction theory (NCT) as a framework for explaining how sociocultural and transcultural factors (including affordances) affect each other in the shaping of landscapes and the evolution of human beings. This approach leads us to an integrative explanation of landscapes as the result of the interaction between human and environmental elements, hence transcending the nature–culture dichotomy.

Before starting to develop our main idea on how to overcome the nature–culture dichotomy thanks to ecological psychology and NCT, it is important to discuss another important aspect of this paper: the idea of landscape. We should first address the different understandings of landscape in the literature in order to clarify what we mean by ‘landscape.’ There are, at least, three different ways to understand landscapes and we will work through the three of them in different parts of the paper. We start by exploring the everyday sense of landscape and its impact on different official definitions by bodies such as UNESCO or the Council of Europe. This sense of landscape is the one that processual landscape theory tries to elucidate. Given that our aim in this paper is to illustrate how landscape theory can benefit from the conceptual tools of ecological psychology and NCT, we also discuss the role of landscape (in the sense of environment or in the sense of the ‘landscape of affordances’ argued for by Rietveld and Kiverstein) within both theories. Finally, we make use of Walsh’s idea of an ‘affordance landscape,’ where the term ‘landscape’ is used somewhat metaphorically, and try to argue that affordances can be incorporated to NCT because the theory can be detached from the adaptationist commitments of some of its proponents.

### Landscape and Perception: Objectivism and Subjectivism

In a captivating paper, Gibson (2001) reflects on the opening scene of John Ford’s *The Searchers* to illuminate the concept of landscape. Of course, you cannot go more “landscapy” than Monument Valley, the quintessential Western landscape, and the awe-inspiring desert view from inside the dark cabin, with just one man riding a horse, makes it even more overwhelming. There is little human impact on these lands. And yet we speak of landscape also when facing less stunning, undomesticated beautiful places. Let us briefly follow Gibson’s lead and refer to the opening scene of a different John Ford movie, *How Green Was My Valley*. Again, the camera starts inside a building, a cottage, and slowly, accompanied by a nostalgic voice, and in one of the most politically poignant camera movements in cinema, Ford leads us to the exterior, to a desolated landscape of smoke, grime, and poor elderly and children, all that the mining industry has left of a once green “and possessed of the plenty of the Earth” Welsh valley. From solitary people riding through deserts to legions of miners trudging toward their jobs, individuals, communities, and natural settings interact in the shaping of landscapes. However, this plural conception of landscapes has not always been the mainstream approach. Gradually, the definition of landscape changed from something considered as natural scenery interpreted from an artistic or pictorial perspective (Jackson, 1984; Pungetti, 1996) to a more comprehensive and inclusive conception (everyday areas).

Despite this progressive development, the mainstream analyses of landscapes have remained committed to a purely cultural approach and to either horn of the objectivism-subjectivism dilemma (Lothian, 1999). In this sense, the cultural perspective permeates the idea of landscape by regarding landscapes as artistic objects (rather than natural ones) with an aesthetic quality. Objectivism claims that landscapes possess an inherent quality that can be evaluated in the same way as we do with other physical features, so that the methodological approach used today relies on different surveys in which individuals evaluate the quality of landscapes based on different assumptions (Lothian, 1999, p. 179). On the other side, subjectivists believe that the quality of a landscape is in the eye of the beholder. They focus on the individual’s taste and capacities to judge the beauty of landscapes, whether using statistical methods or focusing on phenomenology and experiential approaches (Lothian, 1999, pp. 179–180).

There are at least three main problems with this traditional and dualistic approach: first, it is solely focused on a cultural approach; second, it considers landscapes as objects of contemplation; third, it does not take into account the role of humans in the shaping of landscapes. By contrast, we conceive of landscapes as primarily part of our natural environments and, hence, a naturalization of landscape theory is in order. Furthermore, mere contemplation of our surrounding environment is not a good strategy for defining landscape, since we actively interact with it, shaping our landscapes in the process. In this sense, landscapes are to be considered a product of the interaction of humans and their environments, against the contemplative stance.

### Overcoming Objectivism and Subjectivism

Landscapes are conceived of as special kinds of spaces, the product of the interaction of human activities and/or environmental elements: “‘Landscape’ means an area, as perceived by people, whose character is the result of the action and interaction of natural and/or human factors” says the Article 1 of the *European Landscape Convention* (Council of Europe, 2000: Article 1). This co-constitution of landscapes makes them suitable for being explained from a naturalist perspective, analyzing the processes that make them arise and that include human activities as a constitutive element.

The UNESCO definition made the same transition a few years earlier and included another element that we find important: even the most everyday landscape or those minimally affected by the people who live in them are filled with meaning for generations of inhabitants. In its *Operational Guidelines for the Implementation of the World Heritage Convention*, cultural landscapes are defined so:

Cultural landscapes represent the “combined works of nature and of man” designated in Article 1 of the *Convention*. They are illustrative of the evolution of human society and settlement over time, under the influence of the physical constraints and/or opportunities presented by their natural environment and of successive social, economic and cultural forces, both external and internal. (February 1996, paragraph 36; July 2012, paragraph 47)

Landscapes are no longer objects of contemplation, and even *cultural* landscapes include the constraints and opportunities provided by the environment. Furthermore, as Fowler, a former Secretary to the Royal Commission on Historical Monuments (England), highlights, cultural landscapes are to be distinguished from sites of mixed natural and cultural heritage (Fowler, 2006, p. 1). Moreover, while UNESCO is here on the business of selecting outstanding examples of cultural landscape of universal value, a broader conception can be derived from the first sentences of the definition: cultural landscapes need not be profoundly affected by human interaction, as long as they hold meaning across generations of local populations. Fowler points out that the first cultural landscape listed by the UNESCO (Tongarino National Park, in New Zealand) falls into this category (Fowler, 2006, p. 2). Let us finish our reference to Fowler by mentioning an idea from an earlier work of his:

By recognizing ‘cultural landscapes,’ we have, almost for the first time, given ourselves the opportunity to recognize places that may well look ordinary but that can fill out in our appreciation to become extraordinary; and an ability of some places to do that creates monuments to the faceless ones, the people who lived and died unrecorded except unconsciously and collectively by the landscape modified by their labors. A cultural landscape is a memorial to the unknown laborer. (Fowler, 2001, p. 77).

Landscapes are co-constituted by humans and their environment. The environment provides constraints, opportunities, values, meanings, or significances. These are terms that are often used to define affordances, as we will see below. Any attempt at naturalizing landscapes must incorporate both human activities and constraints/opportunities (affordances) as constitutive elements.^1^

Ecological psychology is suited for the task, since it claims that the starting point for understanding psychology is not the individual’s inner processes, but the engagement or coupling between the active organism and the surrounding environment (Gibson, 1979/2015; Richardson et al., 2008). This engagement or coupling starts when agents or organisms detect certain information that guides their actions. In this sense, organism and environment cannot be fully understood separately, much in line with the definitions of landscape quoted above. Ecological psychology starts from the interaction of the organism and some elements of the environment, with affordances being the main objects of perception. Affordances are the opportunities for action that are present in the environment, and agents can perceive them thanks to their exploratory behavior.

The word ‘affordance’ was coined by Gibson (1979/2015, p. 119) as a derivation of the verb ‘to afford.’ An affordance, a feature of the environment that allows us to act in a certain way, is defined as an adjective by using the verb of the action and the suffix ‘-able,’ which reveals that capacity for achieving that action. For example, floors are walkable or cups are graspable. This is the best way to emphasize that affordances are properties of the environment that relate to the organism’s capacities. Now we can understand the suitability of the organism–environment engagement of ecological psychology for the definition of landscape.

Traditionally, affordances are understood transculturally, showing how the anatomic or physiological features of agents (like the shape of our hands), are related to some features of objects (like the shape of a cup) that allow them to perform certain actions (grasping the cup). However, some authors adopt a sociocultural view of affordances, claiming that the variety of affordances expands depending on different normative practices and sociocultural conventions (for example, a computer keyboard affords not only grasping but also typing). We discuss these differences and their consequences more explicitly in section “Niches and Transcultural and Sociocultural Affordances.”

The relation between landscape theory and ecological psychology has been put forward before (Heft, 2010; Rietveld and Kiverstein, 2014). Ecological psychology provides a theoretical and scientific framework that aligns with the idea of landscapes as the product of the interaction of organisms and environment, based mainly on the concept of affordance. Thus, affordances offer a bridge for relating landscape theory and ecological psychology in order to develop a naturalized approach to landscapes, as is the case with ‘processual landscape theory’ (Menatti and Casado da Rocha, 2016). The processual landscape implies a strong notion of agency that comes from the embodied and situated approach of ecological psychology, in which the whole agent perceives the surrounding affordances, seizes them and then helps to create a landscape with his or her own actions (Menatti and Casado da Rocha, 2016, p. 8).

Accepting the explanatory power of affordances leads to challenging deeply entrenched traditional dichotomies in philosophy and psychology, such as subjective–objective (Gibson, 1979/2015, p. 35) or action-perception. Affordances “put meaning back into the world” (Costall, 1995, p. 477) without accepting the main dichotomies that guided cognitivism. In this sense, ecological psychology, thanks to the idea of affordance, transcends the sharp distinctions that guided representationalism and cognitivism and, hence, is capable of overcoming one of the most firmly rooted assumptions in philosophy and psychology: the divide of the natural and the cultural.

In fact, we contend that processual landscape theory, thanks to the adoption of an ecological framework, incorporates most elements for doing this. In fact, we claim that processual landscape theory may benefit even more if it takes in more elements from the ecological approach. Starting from this assumption, our proposal focuses specifically on the nature–culture dichotomy and on how it can be overcome if we go one step further and show how, from an evolutionary approach, this dichotomy proves inadequate for understanding landscape theory within an ecological perspective. This idea has previously been taken into account by Menatti and Casado da Rocha when they argued that “landscapes are the product of the dialectic between culture and the affordances of a place (…) it is a process in continuous evolution (…)” (Menatti and Casado da Rocha, 2016, p. 13). However, they do not develop a specific evolutionary framework, and we aim to fill this gap and to try to solve certain problems and tensions that we find in their proposal by highlighting some important points in ecological psychology, niche construction and their connection.

### Main Idea and Plan of the Paper

Our hypothesis is that, just as the very idea of affordance can serve to overcome the subjective–objective dichotomy, the ideas of ecological psychology and ecological niche, properly defined, would allow us to also transcend the nature–culture dichotomy and, with it, the above-mentioned dilemma between a transcultural and a sociocultural conception of affordances. Our proposal is that sociocultural and transcultural aspects could be systematically accommodated if we apply NCT as a methodological framework for explaining the emergence of ecological niches. This approach will lead us to offer an integrative explanation of landscapes as the products of the interaction between human (both cultural and biological) and environmental elements, making it a clear example of a concept that transcends the nature–culture dichotomy.

In the second section, we explore some implications of relating affordances and the idea of landscape. There we show that different conceptions of affordances lead to different conceptions of niches, which may be unsatisfactory for an integrative account of a landscape. However, we claim that it is possible to account for both natural and cultural elements as fully working together and shaping a landscape if we apply an evolutionary perspective to it via NCT, as we do in section “Integrating Affordances and Landscape through NCT.” According to NCT, organisms modify their niches in such a way that these modifications may lead to certain consequences in the evolution of the organisms that inhabit them. This means that, in shaping a landscape, both cultural and biological aspects affect each other in the course of evolution, according to advocates of NCT. In this sense, this integrative approach is easy to reconcile with the above-mentioned definition of landscape.

## Affordances, Niches, and Landscapes

### Affordances

If landscapes are the product of the combination of human and non-human constituents, then affordances may offer an integrative explanation of their formation and dynamics. Affordances are meant to explain how we perceive and act in a way that makes traditional dichotomies in psychology and philosophy obsolete. A useful example is what happens to the objective–subjective dichotomy: affordances are neither abstract, mind-independent physical features of the objective environment nor purely subjective values; rather, they are environmental properties *relative to* the organisms that perceive them and, as such, they challenge the contrast between subjectivity and objectivity when we explain their epistemological significance (Gibson, 1979/2015, pp. 120–121). In this sense, the very idea of affordance challenges the subjective–objective dichotomy, since the value of the surrounding environment is neither intrinsic to the environment nor in the eye of the beholder. As Gibson himself asserted when he talked about tools:

When in use, a tool is a sort of extension of the hand, almost an attachment to it or a part of the user’s own body, and thus is no longer a part of the environment of the user. But when not in use, the tool is simply a detached object of the environment, graspable and portable, to be sure, but nevertheless external to the observer. This capacity to attach something to the body suggests that the boundary between the animal and the environment is not fixed at the surface of the skin but can shift. More generally it suggests that the absolute duality of “objective” and “subjective” is false. When we consider the affordances of things, we escape this philosophical dichotomy. (Gibson, 1979/2015, p. 35).

The above tool-using example shows what an affordance is in an intuitive way. In our experience of perceiving and taking advantage of an affordance, the organism as a whole, depending on its own exploratory activity, perceives the different opportunities for acting as available in the surroundings. The affordances of the environment afford something to the organism inasmuch as the organism complements the affordance, and vice-versa. There is, then, no sharp distinction between the objective, agent-independent character of affordances and the subjective, agent-dependent character of affordances. This complementarity of the organism’s elements or effectivities (Turvey, 1992) and the aspect of the environment is called the organism–environment (O–E) mutuality (Gibson, 1979/2015, p. 4). This mutuality explains the engagement of organism and environment thanks to ecological information. Organism and environment are engaged and form a system (O–E system) because the organism detects ecological information in the environment that is both necessary and sufficient for guiding its action (Richardson et al., 2008). This detection initiates an engagement between organism and environment manifested as a dynamic loop in which the detection of certain ecological information leads to another action, which in turn modifies the environment, leading to another perceptually guided action, and so forth. Thus, ecological information allows the O–E system to establish itself as the main level of analysis for understanding cognition from an ecological standpoint.

Ecological information is crucial for defining an affordance, because it does not consist merely of the physical forces in the surrounding environment, such as light or sound. As Chemero (2009, p. 107) explains, if an organism is in a fog-filled room, there is light (the physical force), but in that case the light does not reveal or carry information about the surfaces of the room to the organism precisely because of the presence of fog. Thus, the key is the way in which those physical forces *carry certain information about the environment* to the agent. In a room not filled with fog, the light carries information about the surrounding surfaces, and that information is directly detected by the organism. This means that the mere detection of that information is sufficient for guiding the organism’s actions. When the organism detects this information, then it perceives the surrounding affordances (for example, the organism perceives the floor as walkable and the obstacles as avoidable).

In this sense, what we perceive are not absolute measures of the surrounding environment, but relative measures. For example, what we visually perceive when we run toward a wall is not the distance from our bodies to the wall in meters, but the temporal proximity to the wall from an egocentric perspective (Lee, 1976), or what is called the time-to-contact. Thus, ecological information is of a special kind, because it is composed of higher-order variables, among other elements. Notice that they are called ‘higher-order’ or ‘ecological’ because with that term ecological psychologists do not merely refer to the physical structure of the light, but to the light as related to the observer’s capabilities (Gibson, 1979/2015, p. 32). In the case of time-to-contact, we detect the variable tau, which is the ratio of the size of a projected image to the rate of the change of the image’s size, as defined by Chemero (2009, pp. 124–125). Thus, once we detect the variable tau, we perceive certain possibilities for acting (we perceive the avoidability or the possibility of collision, depending on how fast we are approaching the wall). Thus, as we can see, the detection of ecological information is sufficient for guiding our actions, and it allows us to perceive the affordances of the environment. Ecological psychologists define this kind of perception as ‘direct perception,’ since the available information for acting can be perceived without appealing to mental representations or inner information-processing mechanisms (Michaels and Carello, 1981).

To explain direct perception, most ecological psychologists support the Turvey-Shaw-Mace view of ecological information, which states that there is a lawful relation or connection between objects and events in the environment, the changes in the patterns of energy (e.g., sound or light), and what agents perceive due to their action. This lawfulness is characterized as a unique correspondence of environmental features, ecological information, and perception. The idea of unique correspondence is called ‘specificity’ in the ecological approach (Turvey et al., 1981; Shaw et al., 1982). The overall picture is called Shaw’s principle of symmetry, which is the idea that a certain element of the environment specifies certain informational pattern which, in turn, specifies the perception of affordances, and vice versa (Chemero, 2009, p. 111). The symmetry of this principle entails that environment, information, and perception determine each other lawfully (Ibid.). This lawful relation of unique correspondence or specificity guarantees the direct character of perception by grounding it in natural law. In their view, supporters of the Turvey-Shaw-Mace approach are guided by the idea that “there are ecological laws relating organisms to the affordances of the environment” (Turvey et al., 1981, p. 237).

Whether they hold the Turvey-Shaw-Mace view of direct perception or not, all ecological psychologists agree with the idea that the ecological environment of agents is an ecological niche. Here, ‘ecological’ does not refer to ‘Ecology’ as the field of the life sciences. As Gibson himself explained:

A species of animal is said to utilize or occupy a certain niche in the environment. This is not quite the same as the *habitat* of the species; a niche refers more to *how* an animal lives than to *where* it lives. I suggest that a niche is a set of affordances. (Gibson, 1979/2015, p. 120).

The ecological view of niches does not concern specific areas that organisms inhabit, but rather the way in which organisms behave. It refers to the aspects of the environment that allow animals to behave in one way or another and that also depend on the animal’s actions, intentions, and goals. In this sense, a niche in ecological psychology is a set of affordances.

This has consequences for the relation between affordances and landscapes. All ecological psychologists share the idea that we humans modify our environments once we take advantage of affordances, because our surroundings are reconfigured by our impact on them. Thus, the seizing and perceiving of affordances shape ecological niches^2^ and determine the development of the landscape that is a product of the human–environment interaction.

Also, the idea of affordance has clear consequences for landscape theory. Given how this idea overcomes the subjective–objective dichotomy, if we include affordance in our definition of landscape, we conclude that the traditional subjectivist and objectivist approaches to landscape theory mentioned in section “Landscape and Perception: Objectivism and Subjectivism” (Lothian, 1999) reveal themselves as inefficient in explaining landscapes from an affordance-based framework. In the following section, we analyze how the idea of affordance has been defined and how the idea of niche in the ecological approach changes inasmuch as we decide to expand the set of affordances.

### Niches and Transcultural and Sociocultural Affordances

We have discussed how landscape theory has traditionally been approached from the dualistic viewpoint of the subjective–objective dichotomy. However, this is not the only dichotomy in play; rather, landscape theory has also been burdened with a nature–culture split. This latter duality has remained through the years as a purely cultural theory (Menatti and Casado da Rocha, 2016, pp. 5–6).

Traditionally, this nature–culture dichotomy implies that social or cultural practices can be understood without appealing to natural laws, and natural traits and aspects can be understood in isolation from cultural norms. There have also been approaches aimed at subsuming one aspect under another or that have even tried to deny one of the two elements (e.g., Ortega y Gasset, 1929; Mead, 1935; Pinker, 2002), but few have tried to transcend this dichotomy altogether. One of these approaches was ecological psychology. As Gibson stated:

It is also a mistake to separate the cultural environment from the natural environment, as if there were a world of mental products distinct from the world of material products. There is only one world, however diverse, and all animals live in it, although we human animals have altered it to suit ourselves. (Gibson, 1979/2015, p. 122).

**FIGURE 1:**
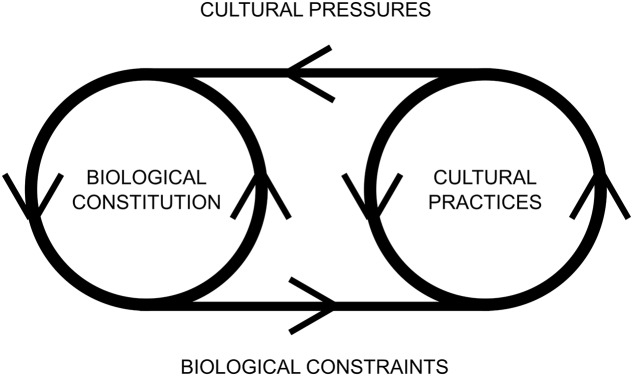
Relations between our biological constitution and our cultural practices. Cultural practices exert some pressure on our biological constitution while our biological constitution constrains the range of possible cultural practices. The two shape each other from an evolutionary approach.

Thus, ecological psychologists hold that, along with the objective–subjective dichotomy, another division that must be surmounted is the nature–culture dichotomy. Thus, overcoming of the nature–culture dichotomy is a goal both for the definition of landscape that we follow and for ecological psychology, which helps to strengthen the above-mentioned similarities between the two ideas. We share that goal, and we view our cultural practices as well as our biological nature to be so entangled that they cannot be considered as isolated elements inasmuch as our cultural norms and our biological processes share the same environmental space (Rietveld and Kiverstein, 2014, p. 329). The question is, then, how do they relate to and affect each other? We propose that this relation is a bidirectional one, as it is shown in **Figure 1**.

In this picture, our cultural practices as well as our biological nature or constitution appear to be essentially dynamic. Both aspects are constantly evolving, although a supporter of the nature–culture dichotomy may claim that they evolve for different reasons: our biological nature is evolving due to purely biological laws (mainly because of natural selection), while our cultural practices evolve thanks to changes in our social norms and cultural conventions. However, it is incongruous to think that they do not interact with each other if they share the same space and if they are developed through the same organisms. There are reasons to believe that this separation of bodily skills or biological capabilities and cultural variants is not as strict as it may seem (Ingold, 2000/2011, p. 5). On the one hand, our biological constitution imposes certain constraints to our cultural practices. If we recall the use of tools, for example, the plethora of actions that we can perform through them is limited by our own constitution. For example, the design of tools for similar purposes changes drastically because of biological restrictions, as it happens when primates and birds use tools for eating insects. On the other hand, our cultural practices exert certain cultural pressures on our evolutionary development (see some examples of this in Laland, 2008).

As this initial approach shows, there is a mutual influence of cultural practices and biological aspects. This has a strong impact on the conception of a niche. From an ecological standpoint, we argued above that a niche should be interpreted as including the set of affordances available for a given population (Chemero, 2009, pp. 147–148). However, there is no consensus on how to conceive of the role of affordances in their niches in relation to sociocultural practices. For example, some authors (mainly, the ones that support the Turvey-Shaw-Mace view) adopt a transcultural approach, focusing on how aspects common to all humans combine with environmental elements regardless of cultural factors (Gibson, 1979/2015; Turvey, 1992). This transcultural approach has also been called ‘structural,’ since it focuses on body-scaled structures (Heft, 2003, p. 157). In this view, the Gibsonian idea that “there is only one world,” cultural and biological at the same time, is based only on the precept that, regardless of cultural practices, all organisms are lawfully related to different affordances via Shaw’s principle of symmetry. In this view, specificity is key and there is a 1:1 lawful relation between the ecological information available and the perception of an affordance. Thus, cultural practices would simply fall outside the picture.

On the other hand, a sociocultural approach would take into account not merely that social norms and habits somewhat skew our dealings with affordances, but also that social norms expand the variety of affordances in each niche (Costall, 1995, 1999; Heft, 2001, p. 134; Heft, 2007; Rietveld and Kiverstein, 2014, p. 345; Ramstead et al., 2016). In this view, cultural practices exert pressure on the seizing of affordances, but these authors are also open to expand the varieties of affordances available beyond the ones accepted by the Turvey-Shaw-Mace view. As Chemero points out, there are some cultural conventions that, in a certain sense, expand and enrich the informational sources available. For example, in a normal situation in which there is an illuminated room with a beer can on a table, the light carries information about the graspability or the reachability of the can, but our cultural conventions show that the light also carries information about the presence of beer (see Chemero, 2009, p. 119).

This idea has drastic consequences for affordances because, in the Turvey-Shaw-Mace view, the presence of affordances is guaranteed by the presence of ecological information by natural law. According to Chemero, this kind of situation places a strong challenge for the purely transcultural view of affordances, because the relation between environment and perception is not bi-directionally symmetrical; that is, while it is causal from the environment to the organism, it is normative (culturally, conventionally informed) from organism to environment (Chemero, 2009, p. 122). In this sense, Chemero’s approach includes cultural conventions as a key for perceiving affordances that opens the door for an enriched variety of the available affordances in our landscape.

Another example of how to relate the ecological approach to conventions is offered by Golonka (2015). According to her view, the perception of affordances is tied to the existence of lawfully specifying ecological information, so the concept of affordance cannot be generalized to cultural cases. In this sense, she defends a transcultural view of affordances. However, she believes that conventions or conventional information can be understood within an ecological framework (Golonka, 2015, pp. 236–238, 248). According to her, this move allows us to “maintain rigorous definitions of affordances and direct perception, suitable for underpinning action control, while still expanding the ecological study of behaviors into those that rely on conventional information” (Golonka, 2015, p. 236). This is so because, according to Golonka, conventional information also includes elements that are similar to those of law-based ecological information, such as validity or reliability: in fact, from a first-person perspective, the perception of conventional information is also direct and learnable (Golonka, 2015, pp. 240, 242, 248). However, despite the similarities, there are differences between conventional information and ecological information even at this point, as she explicitly claims (Golonka, 2015, p. 140). Take, for example, the following quote:

[I]nformation about affordances is more valid and reliable than conventional information (…) The relationship between the waggle dance and food location is very valid and reliable, *but it is also subject to change* (…) Unlike law-based information, detecting a *conventional information variable is no guarantee that some state of affairs in the world is true*. The honeybee can see a blue card, but the experimenter hasn’t put out any food. This means that *there is not a lawfully defined scope within which conventional information is valid*. It is also possible for conventional information to persist while some state of affairs in the world blinks in and out of existence (the experimenter puts out some food but then takes it away). (Golonka, 2015, pp. 240–241, emphasis added)

There are also authors like Withagen and van der Kamp (2010) that challenge the idea of specificity within the ecological approach, claiming that the idea of ecological information should be expanded given that organisms do not always rely on specific information in order to perform certain actions (Withagen and van der Kamp, 2010, pp. 149, 150, 152–153). According to the authors, the detection of the same variable may lead to the perception of different properties of the environment. Also, animals can perceive the same environmental property on the basis of different variables (Withagen and van der Kamp, 2010, p. 155). In order to avoid these problems, the authors aim at finding a different definition of information that makes justice to the main principles of the direct theory of information (Withagen and van der Kamp, 2010, p. 156). They believe that this can be achieved by defining information in a relational way, but “[n]ot in terms of the relation between a pattern in the array and the environment (as Gibson and Chemero did), but as a relation between this pattern and the perceiver” (Ibid.).

A particularly convenient relational approach to information can be found in developmental systems theory. As Withagen and van der Kamp claim, several authors made a connection between ecological information and developmental systems theory before (Thelen and Smith, 1994; Ingold, 2000/2011), but they emphasize the importance of developmental systems theory with respect to the conceptualization of information. In particular, they claim that Oyama’s concept of ontogenetic information can help to reconceptualise perceptual information (Withagen and van der Kamp, 2010, p. 156). This approach to ontogenetic information claims that, instead of considering ontogeny as a genetically determined process in which developmental information residing in the genes specifies the ontogenetic process that gives rise to a particular form (hence the form already exists in the genetic program), developmental information is not guided by a central (genetic) controller: it must include several different factors none of which is in control of the whole process and, for this reason, animal form is continuously generated rather than programmed. Those factors are, according to Oyama, oversimplified in the traditional ontogenetic view as they are divided in genetic and non-genetic, without taking into account the complex interactions between factors and levels during the whole process. In this sense, what have been treated as non-genetic factors are, according to this new view, as constitutive as the genetic ones. According to Oyama, gene and environment are mutually dependent. The factors of the ontogenetic processes not only interact among them, but also determine and define each other, having no meaning in themselves and only acquiring it in their relations (Withagen and van der Kamp, 2010, pp. 157–158).

Based on this idea, the authors propose a new understanding of ecological information based on developmental systems theory, taking Oyama’s (1985/2000) account of information as a starting point: in their view, the informative character of information does not solely rely on the ambient array, but on the relation between informational patterns of the array and perceptual processes (Withagen and van der Kamp, 2010, pp. 149, 159–160). This is shown by the authors by means of three main points: (1) perceptual information is not reified (it cannot be equated with patterns that reside in the ambient array); (2) perceptual information is defined relationally (because “just as the information that is conveyed by a chromosomal form depends on the ontogenetic process, what perceptual information a pattern in the array conveys depends on the perceptual processes” Withagen and van der Kamp, 2010, p. 158, so a given pattern in the ambient array gets its meaning by relating to the perceptual process in which it participates); (3) it implies a shift of focus on the animal-environment system (because exploratory behavior not only contributes in creating and detecting a variable: it has a formative or constitutive function in determining the perceptual information the variable conveys, which is quite different from claiming that we can understand perception and action by merely examining the information in the array) (Withagen and van der Kamp, 2010, pp. 158–159). Thus, the rationale of the authors for understanding ecological information is parallel to that of Oyama’s (1985/2000) genetic information: information is not ontologically pre-existing and manifests in the particular developmental (ontogenetic or cognitive) process, it is neither in the organism nor in the environment; rather, “it emerges in the developmental process” (Withagen and van der Kamp, 2010, p. 157).

Thus, this idea that Withagen and van der Kamp (2010) take from Oyama (1985/2000) fits very well with our own approach. As we will see in section “Integrating Affordances and Landscape through NCT,” there are many kinds of information that arise in the organism–environment interaction. As Withagen and van der Kamp (2010) note, Gibson’s (1979/2015) and Oyama’s (1985/2000) notion of information are quite similar, and this makes it easier to relate the information of developmental, ontogenetic processes to ecological information in order to find certain continuity between cognitive and biological phenomena.

However, how much we should expand the presence of affordances not based on natural laws is a serious issue from an ecological perspective, precisely because the ecological approach is based on a new ontology (ecological information, specificity, direct perception, etc.) that aims to challenge and replace the cognitivist paradigm in the cognitive sciences. If we expand the notion of affordance to refer to merely cultural conventions (not based on elements of its scientific ontology, such as the specificity of natural law), we should at least consider whether this move trivializes the very notion of affordance and, thus, if this accommodates the idea of affordance within a cognitivist paradigm, precluding the cognitive revolution expected by most ecological psychologists.

On the other side, defenders of what we call the sociocultural approach charge, as does Chemero (2009, p. 112), that if the information for guiding action is that pointed out by the Turvey-Shaw-Mace view, then there is too little information to perceive. However, this is quite surprising because Gibson contended that our environment is full of information for guiding our action. Take, for example, this illustrative situation:

[I]n everyday contexts, body-scaling considerations may be necessary but are not sufficient information for specifying the affordance properties of an environmental feature. (…) Indeed, in his discussion of the information for affordances, Gibson (1979/2015) suggested that something he called “a compound invariant” might be needed for describing an affordance, but he was by necessity quite vague about what this might entail. (…) Stated differently, in most cases the character of the surface has a sociocultural dimension as well as a structural one. Take the case of a step that is memorialized for some sacred or historical reason by a culture. The body-scaled properties of the step might indicate that it could be stepped up on, but all the while it ought not be for reasons quite apart from those factors. (…) My view is that we should continue to push for the perceptual explanation even for such more elaborated considerations of meanings as those grounded in sociocultural processes. (Heft, 2003, pp. 157–158)

These differences in the conception of affordances result in competing views of ecological niches and also in different ideas on how to understand landscapes. In the transcultural view, niches would accept only affordances whose perception is purely based on specificity and natural law. We also considered a different case, one in which there are cultural pressures that force us to take advantage of one affordance over another, integrating the Turvey-Shaw-Mace view on affordance perception within a normative background of cultural practices (Heft, 2003, p. 158; Heras-Escribano and de Pinedo, 2016, p. 587). Finally, according to the sociocultural approach to affordances, purely cultural and not specificity-based or law-based affordances enter the scene, enriching the plethora of affordable moves in our environment, and widening the variety of affordances in our niches (Rietveld and Kiverstein, 2014, p. 326). Regarding these differences, we can enumerate at least three kinds of scenarios in which we perceive and take advantage of affordances:

• Situations in which the perception of affordances depends solely on the active exploration of the environment and the way the environment specifies body-scaled and action-scaled affordances, all based on natural law. Example: the graspability of a cup, the step-on-ability of a step, the avoidability of an obstacle.
• Situations in which the perception of affordances depends solely on the active exploration of the environment and the way the environment specifies body-scaled and action-scaled affordances based on natural law, but including cultural pressures that force us to select one affordance from among others. Example: in a football game, perceiving both the opportunity for passing a ball and that for kicking to score, and passing the ball instead of kicking to score due to social, normative reasons (Heras-Escribano and de Pinedo, 2016, p. 587).
• Situations in which the perception of the (alleged) affordances also includes cultural conventions established by humans so that these affordances do not depend on ecological specificity or natural law. Examples: perceiving that a postbox “affords letter-mailing to a letter-writing human in a community with a postal system” (Gibson, 1979/2015, p. 130); perceiving the drinkability of beer when we see a beer can; the idea of emotional affordances (Hufendiek, 2016; Vallverdú and Trovato, 2016).

The acceptance of these situations and the acknowledgment of transcultural and sociocultural affordances critically changes the idea of environmental niche, and also the relation between our cultural practices and our biological nature. This issue is not trivial: some ecological psychologists would be reluctant to accept purely sociocultural affordances because they would diminish the power and significance of the ecological, law-based explanation of perception and action. Since we agree that “agency means physical perception (we perceive through our body and its movements in space) but it implies at the same time the social/cultural creation of place” (Menatti and Casado da Rocha, 2016, p. 13)^3^, we ask ourselves whether there is a framework or strategy to help us unify the two aspects, relieving the tension between the two views, and justifying the definitions of affordance and landscape offered above. We propose that an elegant way to do this is through the idea of NCT.

## Integrating Affordances and Landscape Through NCT

### Niche Construction and Evolution

Niche construction is the process by which organisms modify their selective environments (Odling-Smee et al., 2003; Laland et al., 2016). These modifications can be beneficial for them and for their offspring and, when this is so, both the gene and the modification to the niche are inherited. NCT aims to explain how the dynamics between cultural aspects and biological aspects over time have evolutionary implications.

This last claim is sometimes taken to be controversial. The mainstream gene-centered approach or gene’s point of view to evolution comes through a particular definition of the idea of adaptation, which is the main term in evolutionary biology. ‘Adaptation’ comes from the Latin word *adaptare*, which divides into *aptare* (to fit) and *ad*- (‘to’). The etymology of the word is quite revealing in this case, because it means something like ‘to be suited to something,’ which implies the existence of a previous world to which organisms must fit if they seek to survive, and this is the way in which defenders of the gene’s point of view interpret adaptation. In this view, selection occurs in response to facing the selective forces, pressures, and effects of natural selection (Lewontin, 1978, 1985; Sultan, 2015, p. 36). Adaptation is defined as an asymmetric process because an external force (natural selection) is the one that drives evolution and organisms are either suited to the environment or not. If organisms survive the environmental conditions because they have certain genetic material, they reproduce and pass their genes to their offspring. This gene-centered version is the mainstream view of adaptation in the neo-Darwinian perspective, also known as the view of the Modern Synthesis, the product of the synthesis of Darwinian natural selection and Mendelian genetics (Huxley, 1942). Thus, the genetics of populations or species is the main level to focus on, since genes are what pass the filter of pre-established and pre-existing environmental circumstances. This view has certain ramifications: organisms are passive receptacles of genes, the only force that drives evolution is natural selection, and inheritance is only genetic.

However, niche construction is a process that follows a slightly different logic to that of the gene’s view and aims to be vindicated as having a role in the drive of adaptation and evolution. In this view, organism–environment reciprocity is key for establishing dynamics that bias the direction of selection. Thus, while most biologists consider natural selection to be the major (or even the sole) force that drives evolution, some postulate that niche construction is a process that can bias the direction and rate of selection within certain populations. In this sense, defenders of niche construction are not against the idea of the centrality of adaptation: they simply maintain that niche construction plays a role in adaptation at many levels. “Hence NCT recognizes natural selection and niche construction as reciprocal causal processes in evolution, and treats the adaptations of organisms as products of both processes” (Kendal et al., 2011, p. 785). This has historical antecedents in the literature. Some historians and philosophers of biology claim that the Darwinian project of explaining evolution was based not only on natural selection, but also on the ecological or mutual dynamics of organism and environment. In their view, Darwin aimed to explain, for example, how the behavior of the organism was adaptive from an ecological point of view (?, p. 193; Reed and Jones, 1977, p. 153), this being especially central in his works on plants and minimally cognitive animals (Darwin, 1880, 1881). Even one of the founders of the Modern Synthesis claimed that adaptation was a kind of harmony between organism and environment (Dobzhansky, 1937, p. 170; Sultan, 2015, p. 35). In order to understand this so-called harmony between organism and environment, it is important to focus on the organism as a whole: the phenotypic expression of genes and its behavior. Thus, the gene’s point of view is not the only level for explaining adaptation. This is why niche construction is a force of interest, because it includes certain symmetry in the relation, since organisms are capable of shaping or constructing the external conditions of their existence (Lewontin, 1985; Odling-Smee et al., 2003; Sultan, 2015, p. 37). There are two main ways of shaping the external environment: through perturbations (changes in organismal behavior that physically alter environmental variables) or by relocations (movements in space that encounter different sets of variables) (Ibid.). Thus, niche construction is a symmetrical process because organisms shape their environments that, in turn, impose certain selective pressures on them.

Within the field of NCT, a recent development has emphasized the role of social niche construction for cultural species. The focus tends to be on the production of social structures that facilitate survival for their members. For instance, explanations of cooperation often highlight the existence of population structures where cooperators can benefit from each other’s actions and social niche construction provides an explanation of the evolution of such structures (see Powers, 2010; Powers et al., 2011). This is particularly significant in the case of affordances: sociocultural affordances necessarily include explanatory, mediational, and cooperative factors in order to take advantage of them. NCT helps us understand sociocultural affordances in a naturalistic way, taking into account our sociocultural dimension as being also constitutive or formative of our organism–environment system (Heft, 2007), since the contribution of our sociocultural dimension in terms of affordances is critical when offered within an evolutionary framework. The evolutionary drift of human beings, for example, could never be fully understood without appealing to our sociocultural factors, and introducing sociocultural affordances help us to understand this evolutionary drift of organism and environment in a co-constitutive and reciprocal way regarding our environment. Thus, the key to our sociocultural nature lies on our mediational and cooperative aspects, which are constitutive parts of the sociocultural affordances, because this kind of affordances appears within a sociocultural background of norms and conventions (keyboards afford typing to societies in which typing is a well-established social practice). This has consequences for landscape theory: in this way, sociocultural aspects are easily included as formative aspects within a naturalistic framework that explains the evolution of landscapes, just like processual landscape theory demands (Menatti and Casado da Rocha, 2016, p. 14) (see section “The Engagement of Nature and Culture from an Ecological and Evolutionary Perspective” for a discussion of the more intricate relation of NCT and processual landscape theory). At the same time, these mediational aspects in which organisms teach or indicate some others the proper way to take advantage of an affordance may be applied to transcultural affordances as well, as we will see in section “Affordances and Evolution.” However, moving beyond the coevolution of cooperation and population-structuring behavior, it can be argued (see Sinha, 2015) that language and other symbolic dimensions of cultural communities are social niches in the same sense as population structures are. This suggestion goes much in line with our insistence at the beginning of the paper on the centrality of the meanings and values that landscape may embody for generations of inhabitants. Viewing language as a kind of socially constructed niche goes a long way into incorporating such symbolic dimension.^4^

Typical examples of niche construction include both sociocultural and transcultural aspects: for example, lactose tolerance in humans has been benefited by the adoption of dairy farming, a practice that exerted selection pressure in evolution, favoring a dynamical feedback between that sociocultural practice and the allele for lactose persistence (Scott-Phillips et al., 2013, pp. 1233–1234). Another example concerns beavers living in a flat landscape with a river running through it. The beaver’s genotype includes genes that code for a whole range of behavior related to cutting down trees with their teeth and dragging logs around to construct dams. That changes the landscape (dams the river, forms lakes) and thus has constructed a new niche with a whole new set of affordances for the beavers. The next generation of beavers will fare better because they now live in a swampy lake environment rather than an open forest next to a river. Thus there’s selection for the relevant genes via their action on the beaver environment.

Thus, given that niche construction is guided by (different kinds of) information driving environments into different and unexpected states (Scott-Phillips et al., 2013, p. 1234), we propose that ecological information for the perception and taking advantage of affordances may fit this definition, as well as certain other kinds of sociocultural practices that exert pressure for seizing affordances. With this strategy, we can integrate both the transcultural affordances as well as sociocultural ones, showing how the mutual reciprocity of biological form and cultural practices (see again **Figure 1**) overcomes the nature–culture dichotomy from an evolutionary and ecological standpoint. This combination of the ecological approach and NCT is consistent with the principles of processual landscape theory, which also includes affordances and ecological principles.

### Affordances and Evolution

Although niche construction has previously been related to ecological psychology to overcome the nature–culture dichotomy (Heft, 2007; Withagen and van Wermeskerken, 2010), we posit that there is more work to be done stressing the *evolutionary* character of affordances within niche construction. In particular, the evolutionary consequences of our perturbations, i.e., the behavioral shaping of our environment thanks to our perceiving and taking advantage of affordances. Although advocates of NCT consider cultural niches to be different from environmental ones, the former are still a subset of niche construction that is the expression of culturally transmitted knowledge (Odling-Smee and Laland, 2012, p. 226). As such, both inherited aspects affect each other in the process of niche construction. This is how cultural conventions as well as social norms are mutually affected by purely biological traits: in the mutual, causal bi-directionality of environmental and organismal evolution.

A key aspect that is useful for relating ecological psychology and evolution emerges from the idea of ecological inheritance, the material consequences of niche construction processes (Odling-Smee et al., 2003). As we have seen, the results of the shaping process of niche construction are inherited through generations. In the case of humans, too, those inheritances (cultural and non-cultural objects of our environments that may have the shape of shelters, tools, culturally loaded material objects, etc.) are not genetically but socially transmitted by organisms within and between generations through modifications of the external environment (Odling-Smee and Laland, 2012, p. 223). In relating affordances and niche construction, the idea of ecological inheritance is key for illuminating the evolutionary role of affordances, because, together with the ecological inheritances themselves, the function of the structure is maintained within and between generations.

Our approach to this idea of ecological inheritance is modified by the inclusion of affordances in the evolutionary picture. Furthermore, if these affordances are key for guiding and constraining human organismal behavior, it is presumed that: (1) humans are helped and taught by their mates to educate their attention to detect specific information that can guide action (Gibson, 1950, p. 155; Costall, 1995, p. 477; Ingold, 2000/2011, p. 36); (2) the perception and taking advantage of different affordances (either transcultural or sociocultural) is affected by the normative and cultural environment in which every human is embedded, and in some cases those social norms press for taking advantage of one affordance instead of another (see section “Niches and Transcultural and Sociocultural Affordances,” as depicted in Heras-Escribano and de Pinedo, 2016, p. 587). We conjecture that this works for all kinds of ecological inheritances, be they cultural or not. For example, we might think of culturally informed structures that are seized by the offspring of a population, such as architectural spaces (schools, churches, or sport venues) or tools; all of them serve different social functions, and the behavior we may deploy is considerably different. As such, different cultural conventions and social norms educate our attention and sensitivity in order to be receptive to certain affordances or ecological variables that help us guide our actions when we deal with those elements. Also, we can think of the perception of different non-cultural scenarios, such as when humans explore a new territory, hunt or fish (Ingold, 2000/2011, p. 36). In all these scenarios, there are both affordances and cultural conventions. In the picture we are sketching, in which there is a mutual and reciprocal influence of cultural conventions and ecological information, the distinction between cultural and natural aspects blurs since both kinds of aspects contribute equally to organismal and environmental evolution.

The key point is that what is transmitted through ecological inheritance, the material consequences, are not just the structures (buildings, tools, or non-culturally informed environmental elements), but also the social functions of those structures together with the behavioral strategies that help us deal with them, and affordances are key for explaining those strategies. These behavioral strategies are inherited through social mediation, in which the community or social environment teaches its members what to do or what to attend to. This is why the function of the structure is maintained within and between generations (tools and everyday objects, for example, have a function maintained by instruction and social reinforcement). If the function of the structure is maintained, this means that the role of the structure is similar; and, for this similarity to be achieved, the ways of dealing with the structure should be identical^5^. For example, in practices such as hunting and fishing, the material consequences that are ecologically inherited along with tools and weapons are also the behavioral patterns for hunting and fishing, which result from the socially educated attention to the affordances that those tools and weapons offer us.

**FIGURE 2:**
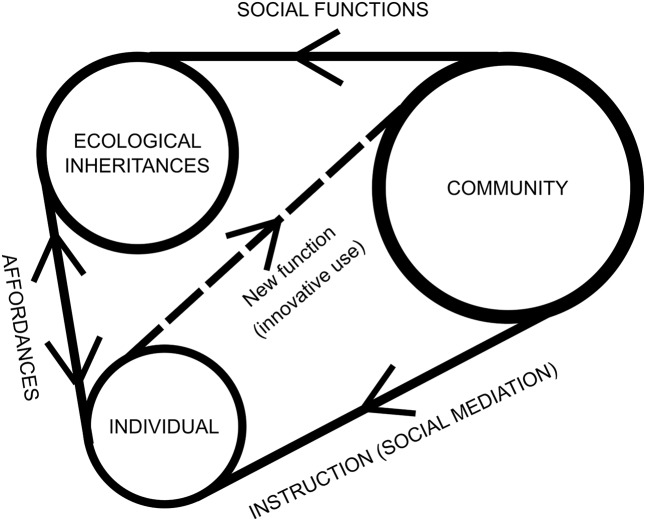
Different interactions among an individual, her community and the ecological inheritances of her environment. Ecological inheritances are bestowed by the community with social functions that are taught to individuals via instructions, and those instructions serve to deal with the affordances available to individuals. At the same time, an individual can engage with an innovative use of an affordance and transmit it to the community (dotted line). If accepted, a new function for ecological inheritances is established.

**Figure 2** shows all relations in a systematic way.

In this sense, what a structure affords to the agent depends on the way the agent explores the environment and also on the way the affordances of the structure modulate the subsequent behavior of the explorer, allowing certain actions to be undertaken or precluding them. This dynamic process of exploring, perceiving, and acting is the basis for the engagement of the agent with a structure, and if the product of this dynamic engagement results in a certain benefit for the organism, its population, and/or the offspring, this establishes a function that is inherited along with its affordances.

### Landscape, Affordances, and Evolution

The above sketch, which relates ecological psychology and an organism–environment mutuality from an evolutionary perspective, has clear consequences for processual landscape theory. In fact, it is not strange to find similarities between a theory of landscape and different usages and understandings of landscape. Take, for example, Walsh’s (2014a) proposal of an *affordance landscape*. Walsh develops, in a way that fits with our claims, his idea of an affordance landscape as an alternative to the adaptive landscape of adaptationism. In his view, an affordance landscape reacts against the gene-centered view of adaptationism and stresses the role of the organism and the symmetry of organism and environment. We believe that this kind of approach can improve Menatti and Casado da Rocha’s processual landscape.

Walsh argues against proponents of the adaptive landscape and he offers his own approach, the affordance landscape. Proponents of the adaptive landscape^6^ maintain that biological form and landscape are, as we have seen in section “Niche Construction and Evolution,” asymmetrically related: form evolves in response to landscape, but not vice versa (Walsh, 2014a, p. 216). These proponents claim that “the environment is largely autonomous with respect to the organisms” and also that it works “according to its own intrinsic dynamics” (Godfrey-Smith, 2001, p. 254). This makes the adaptive landscape something inert and unchanged, like a mere container with its own autonomy, separated from the organisms that populate it. However, landscapes are also the products of the interaction of human activities and environmental elements because the features of the affordance landscape are influenced by the biological form or constitution of the organisms that occupy it. In this sense, Walsh takes the relation between affordances and organisms to be symmetrical. As it may seem, Walsh’s approach is focused solely on transcultural affordances. In order to illuminate this, Walsh appeals to the phenomenon of phenotypic plasticity (West-Eberhard, 2003; Walsh, 2014a, p. 230).

Phenotypic plasticity is the ability of an organism to compensate for an external perturbation by changing a feature of its phenotype, which leads to a cascade of changes throughout its phenotype, given the high grade of functional integration of different phenotypic aspects (West-Eberhard, 2003, p. 98). This integration is called phenotypic accommodation, a mutual adjustment of phenotypic variations without genetic change:

For example, the increase in the mass of a muscle in response to the demand for greater force also requires changes in the origin and insertion of the bones. It further requires increased vascularisation, innervation, and changes in associated connective tissue. (Walsh, 2014a, p. 227)

These compensations for taking advantage of a demand or an affordance, in turn, alter the environment through the action of organisms. If we understand biological phenomena such as phenotypic plasticity under the light of ecological psychology, we find that it is a goal-directed capacity for responding to the affordances of the environment (Walsh, 2014a, p. 228). Sometimes, these responses are inter-generationally stable (Ibid.), which means that the evolution of landscapes and organisms is a product of their co-established dynamics, as we have seen in the previous section. In conclusion, authors like Walsh maintain that the notion of affordance is not only key for explaining evolution, but also for understanding landscape as a product of the interaction of the dynamics established by organisms and the environmental elements.

However, while we have grounded our evolutionary approach to affordances in niche construction precisely because niche construction shares with affordances the notion of organism–environment mutual shaping, Walsh criticizes the idea of niche because he takes it to be an example of how the adaptive landscape explains evolutionary processes. He claims that “[a]n evolutionary niche is a set of properties of an organism’s environment, to which organismal form may fit (…) [o]rganismal form and the niches to which it adapts are decoupled and asymmetrically dependent” (Walsh, 2014a, p. 217). We believe that this idea of niche is not the only possible one, as we have seen thorough the paper. In fact, the idea of a niche as the asymmetric environment of the adaptive landscape has been modified since it was initially conceived to include the idea of niche as a construction of the organism (Lewontin, 2000, p. 48), hence including the symmetry demanded by Walsh. In fact, NCT claims that organisms do not just fit into their environments, but they modify their environment, just as Walsh claims, and “[w]hen such modifications alter natural selection pressures, evolution by niche construction is a possible outcome” (Laland et al., 2016, p. 192).

This last sentence of Laland et al. (2016) could be understood as if some form of the asymmetry criticized by Walsh could be at work, but this cannot be the case. As we understand it, the environment at all its levels is co-constituted by the actions of organisms (as it is common in ecological psychology, processual landscape theory, our understanding of NCT, and Walsh’s affordance landscape). This means that the way the environment affects organisms is not autonomous with respect to the action of agents, precisely because the way it affects agents has been already modified by the actions of those organisms themselves. If one claimed that this active shaping of the selective conditions is a mere irrelevant or non-constitutive aggregate, it would be a proper target of Walsh’s criticism and it would be committed to the asymmetry, given that the actions of agents (or the lack thereof) would not modify the identity of the environments. In contrast, we take those actions to be constitutive of the identity of the environment organisms are part of, so this constitutive character necessarily implies symmetry. Thus, niche construction is a process that may be conceptualized as one of those factors that Oyama (1985/2000) mentions as having a constitutive or formative role: organism and environment reciprocally affect each other in their mutual development, and those affections are always determined by the previous ones, which makes every interaction constitutive or formative of the following affection. Having all this in view, the idea of ‘selection pressure’ itself does not necessarily imply an asymmetric commitment if it is understood from the constitutive perspective we are introducing in these lines.

There is also another aspect in our approach that emphasizes the symmetry proposed by Walsh in his affordance landscape: the affordances of the niche are fitted to the organism’s goals and purposes, which are a reflection of its form. This means, as Walsh claims, that “adaptive evolution arises from organisms’ purposive interactions with their conditions of existence” (Walsh, 2015, p. 169). In this picture, “[t]he conditions in which form evolves are a joint project of the organism and its setting” (Ibid.), and the best way to explain this joint project and reciprocity of environmental elements and organismal form is the idea of affordance. This is so because, when an organism acts upon its own environment by means of taking advantage of an affordance, it creates new affordances: these affordances shift as form changes (Walsh, 2015, p. 174). Our view of NCT is fully in line with these ideas, as it includes the mutual shaping of organism and environment in a constitutive way, achieving the symmetry demanded by Walsh’s affordance landscape against the asymmetry of the adaptive landscape.

There is another aspect of Walsh’s affordance landscape approach that we want to discuss: although it includes ecological elements such as affordances and evolutionary elements such as phenotypic plasticity, it leaves aside the essential role of cultural conventions and social norms that the processual landscape theory includes for explaining landscapes. In fact, Walsh neither discusses the difference between the Turvey-Shaw-Mace view and the possibility of sociocultural affordances nor analyses the influence of cultural conventions in his proposal of the affordance landscape. A plausible explanation is that Walsh may not be interested in these kind of aspects and merely aims to focus on evolutionary debates. In the same vein, processual landscape theory does not include an evolutionary framework for integrating the dynamic feedback of cultural norms and ecological elements explained in **Figure 1**. As such, we argue that Walsh’s affordance landscape and the processual landscape theory can reciprocally benefit, since they include aspects that are key for each approach (the affordance landscape includes evolutionary change while the processual landscape includes the cultural dimension).

Only if NCT needed to share the asymmetric view that Walsh finds in the Modern Synthesis, our ecumenical proposal would fail. In another paper (Walsh, 2014b), his diagnostic is that there is an unwarranted asymmetry between the role of genes and that of behavior, development, niche construction or learning: only genetic change, change in the relative frequency of gene types, is evolutionary change. NCT, according to Walsh, inherits this assymetric view of evolution. Although this seems the case historically (Walsh offers textual evidence from Laland et al., 2003; Sterelny, 2009), we believe that the way we have introduced niche construction as an integration of affordances and landscapes is far from embracing the “two spaces and a barrier” image of evolution. Despite the reservations of some authors (for instance, Magnani and Bardone, 2008; Sinha, 2015, or those in the enactivist tradition, such as Stapleton, 2016), we find sufficient emphasis in ecological psychology on the role of the agent’s behavior in the construction of affordances (see Reed, 1982; Heras-Escribano, 2016; Pinedo, 2016). In fact, one of the most exciting things about affordances is their relevance and significance in considering not merely subpersonal processes but the agent as a whole as related to the environment (Gibson, 1979/2015, pp. xiii, 4, 51, 195). An aspect that facilitates this agential level is that the same affordance is perceived through different perceptual systems: in examples like the perception of climbability in sensory substitution devices, the ecological information for climbability can be perceived either visually or haptically (Travieso et al., 2015). Explaining behavior in terms of affordances is done at the agential or personal level, where the details of the subpersonal or subagential mechanisms that possibilitate such behavior become irrelevant (see McDowell, 1994; de Pinedo and Noble, 2008 for similar views). In our view, niche construction should be understood as one of the ways in which the agent’s behavior construct affordances that will have a direct evolutionary relevance for the descendants of the agent.

## The Engagement of Nature and Culture From an Ecological and Evolutionary Perspective

As seen in the different sections of the paper, the intertwining of sociocultural and transcultural affordances is so tight that it would be strange to separate one from another. Both are opportunities for acting, both are present in the same environment, and both play the same contribution to the co-evolution of organisms and landscapes. This last aspect is especially important for the goals of the paper because, as we see it, the separation of nature and culture in the form of transcultural and sociocultural affordances is dissolved within niche construction processes if the latter are understood in a symmetric way, similar to Walsh’s affordance landscape. This idea is reached by means of another two: (1) on niche construction, both sociocultural and transcultural affordances are treated in the same way (as similar constitutive factors, to use Oyama’s apt phrase) and are considered *as having similar forming or constitutive effects* on the co-evolutionary development of organism and environment, which makes them indistinguishable in kind from an evolutionary perspective; (2) the understanding of sociocultural and transcultural factors as affordances makes them suitable to be treated from an organism–environment view, not focusing on genes or on populations. Both (1) and (2) are quite important for dissolving the nature–culture dichotomy, since in the processes of niche construction of which they are part, in evolutionary terms both aspects contribute on a similar manner: they are formed by the co-constituted dynamics of the O–E system and they also contribute to generate it at the same time through all the process of niche construction.

Once both kinds of affordances are understood under the same evolutionary view, affordances are simply taken as having these forming or constitutive effect, and their sociocultural or transcultural nature does not add anything to the way in which they help to shape the mutuality and reciprocity of organism and environment within their co-evolution. Another aspect to be emphasized is that this is achieved in the way that processual landscape theory demands because it integrates sociocultural and transcultural aspects in an evolutionary perspective (Menatti and Casado da Rocha, 2016, p. 9, **Figure 1**), it focuses on agency in all its varieties (biological, psychological, cultural) (Ibid.), and, more importantly, it emphasizes the co-constitutive aspect of organism and environment in the shaping of landscape and their continuous and evolving nature through niche construction understood as in Walsh’s affordance landscape (Menatti and Casado da Rocha, 2016, pp. 10–14). Thus, for all these reasons, we claim there is no difference between the transcultural and the sociocultural aspects of affordances regarding their evolutionary contribution to the co-constitutivity of biological form and landscape that we offer here.

As Sterelny (2007, p. 721) convincingly argues, unlike contemporary great apes and early hominids, in us humans the distinction between sociocultural competence and ecological competence blurs. In apes, social complexity varies greatly between species, while the demands imposed by their environment are relatively similar for all of them (Byrne, 1997). In our case, interaction with the environment and social interaction go hand in hand (Gibson, 1979/2015, p. 122; Costall, 1995; Rietveld and Kiverstein, 2014). Furthermore, our mechanisms for social and cultural learning are not limited to learning about the cultural and the social. We also learn how to rely on others’ knowledge and expertise about the environment (Gibson, 1950; Costall, 1995; Ingold, 2000/2011). As seen in section “Affordances and Evolution,” the picture we are sketching, in which there is a mutual and reciprocal effect of cultural conventions and ecological information (see **Figure 2**), makes it difficult to disengage the natural and the cultural as two radically different aspects concerning their evolutionary consequences. The two aspects of our human condition are equally essential for our evolutionary development, and we could not isolate the contribution of one from that of the other, since these two dimensions are equally affected via biological constraints and cultural pressures (see **Figure 1**). In this view based on niche construction, a cultural pressure is as selective as a natural one, so this means that, since both are sharing the same environment, it is quite difficult to separate the contributions of each of them to the evolutionary process. To reach this conclusion, affordances have been essential, both the transcultural and the sociocultural ones. This is because, as we claim in section “Affordances and Evolution,” ecological inheritances not only include the material products (e.g., certain structures) and their functions, but also the patterns of behavior and the affordances for dealing with them (sociocultural or transcultural). One conclusion is that the changes in our environmental niches affect our cultural niches, and vice versa (e.g., building schools affects the cultural inheritance of our children, and farming practices impose artificial selection, as pointed out by Odling-Smee and Laland, 2012, p. 228). Thus, we hold that the distinction between cultural and natural aspects blurs since both kinds of aspects equally contribute to organismal and environmental evolution. This is quite analogous to the subjective–objective dichotomy in ecological psychology. It seems that affordances simply do not fit the logic behind traditional dualistic views that guided our explanations of the cognitive, such as subjective–objective or culture-nature.

This has crucial consequences for our conception of landscapes: if they are the product of our dealings with affordances, and if they are transmitted as inherited in niches, then landscapes are also inherited as the product of this interaction in an evolutionary framework. Thus, the idea of Walsh’s affordance landscape strongly complements the idea of processual landscape theory, because from their synthesis we gain an explanation that includes both cultural and natural aspects within an evolutionary framework. This evolutionary perspective that combines niche construction and affordances proves to be the most effective way to offer a systematic explanation of the formation and development of landscapes, because it can accommodate most changes and variations of the landscape as ecological inheritances. As we have discussed, this framework closely fits the definition of landscape as the product of the interaction between human and environmental elements, making it a clear example of a concept that transcends the nature–culture dichotomy.

## Author Contributions

MH-E: wrote the first draft of the paper, made the illustrations. MDP-G: provided substantial advice regarding its structure and substantial contributions for improving different sections of the paper, especially those on landscape theory and the gene-centered view.

## Conflict of Interest Statement

The authors declare that the research was conducted in the absence of any commercial or financial relationships that could be construed as a potential conflict of interest.

## Footnotes

1 The interconnection between natural and cultural elements and its relevance for a proper understanding of landscape can be further illuminated by considering how some features of landscapes encourage certain types of constructions or even some forms of art. For instance, the stone walls dividing farm lands in Ireland, especially striking in the Aran Islands, are an ingenious solution to the problem that stones present to farmers. Moreover, we often hear sculptors (or architects) highlighting the influence that their childhood landscapes and environment had on their work. Henry Moore and Eduardo Chillida are two influential cases at hand. Here, is Moore: “In Yorkshire, in Adele Woods just outside Leeds, there was a big rock amongst many that I called Adele Rock. That influenced me quite a bit. For me, it was the first big bleak lump of stone set in the landscape surrounded by marvelous gnarled prehistoric trees. It had no feature of recognition; no copying of nature—just a bleak powerful form. Very impressive” (quoted in Nudsen, 2013). And here is Chillida, talking about his famous “Wind Combs”: “The sea has been my master and I have learned much from it. When I was young, I would go there instead of going to school. I watched the waves and thought, “Where do they come from?” I did not know I would be a sculptor, but I think that I was already thinking about “Wind Combs.” The place is the origin of the work” (Wagner, 1997). Thanks to an anonymous referee for pointing out to us examples of this type.

2 ‘Ecological niche’ may refer to the set of affordances for a given organism and also to the idea of a habitat in biology. To avoid confusion, in the rest of the paper, we will use the label ‘environmental niche’ to refer to the idea of ecological niches in biology, but including at the same time the sets of affordances for the population that inhabits that setting.

3 As an anonymous referee has pointed out to us, the claim that agency implies physical perception seems too strong, as well as independent from the idea, dear to ecological psychology, that perception involves agency. Although the authors may actually mean the latter (as the parenthesis seems to indicate), we would not reject the more radical reading straightaway. Although this is not the place to develop the connection, Whitehead and other process philosophers have insisted on the pervasiveness of perception in nature, both in living and in non-living beings. To exist is, for Whitehead, to make an effect on other existents. Furthermore, he understands efficient causes in terms of perception (‘prehension,’ in his jargon): an effect perceives its cause by being modified by it. His metaphysics opposes the idea that reality is made up of substances and replaces it with a picture of the world as an assemblage of perceiving and perceived occasions (see Whitehead, 1929, part III, Chap. I). Bensusan (2016, Chap. 3) has convincingly argued, following Whitehead’s steps, that it is central assumption of any monadology that everything perceives. Monadologies like Leibniz’s understand substances as articulated sets of perceived. For Leibniz, substances are defined as units capable of acting (see Leibniz, 1714/1948, paragraph 1). Neo-monadologies like Whitehead’s replace substances by actual entities that are perceived by others and capable of action based on their physical perception. Even if indirectly, we believe that Whitehead’s process philosophy has exerted some influence on processual landscape theory.

4 Thanks to an anonymous referee for pointing out the importance of immaterial heritage for the shaping of landscapes.

5 If there is functional variation, there is also a social impact and behavioral reorganization, but in all cases following the same logic; that is, attending to the ecological aspects and including social mediation and instruction.

6 By ‘adaptive landscape’ Walsh means the consequences of adopting the gene-centered view for understanding landscapes, as we explained in section “Niche Construction and Evolution.” As such, Walsh ends the paper we comment on by proposing a new way of interpreting adaptation and landscape as based on affordances. In this sense, Walsh’s approach goes against the gene’s point of view and its consequences.
